# Grasp posture modulates attentional prioritization of space near the hands

**DOI:** 10.3389/fpsyg.2013.00312

**Published:** 2013-05-31

**Authors:** Laura E. Thomas

**Affiliations:** Center for Visual and Cognitive Neuroscience, Department of Psychology, North Dakota State UniversityFargo, ND, USA

**Keywords:** visual attention, visual processing near hands, power grasp, precision grasp, hand posture, embodied cognition

## Abstract

Changes in visual processing near the hands may assist observers in evaluating items that are candidates for actions. If altered vision near the hands reflects adaptations linked to effective action production, then positioning the hands for different types of actions could lead to different visual biases. I examined the influence of hand posture on attentional prioritization to test this hypothesis. Participants placed one of their hands on a visual display and detected targets appearing either near or far from the hand. Replicating previous findings, detection near the hand was facilitated when participants positioned their hand on the display in a standard open palm posture affording a power grasp (Experiments 1 and 3). However, when participants instead positioned their hand in a pincer grasp posture with the thumb and forefinger resting on the display, they were no faster to detect targets appearing near their hand than targets appearing away from their hand (Experiments 2 and 3). These results demonstrate that changes in visual processing near the hands rely on the hands' posture. Although hands positioned to afford power grasps facilitate rapid onset detection, a pincer grasp posture that affords more precise action does not.

Objects that are within the reach of an observer present unique demands for visual processing. While visual attention allows for the selection of objects anywhere in the environment, only those items within peripersonal space afford immediate interaction, creating a need to integrate visual information with spatial, tactile, and proprioceptive representations. Observers process information near their hands differently than information presented far from their hands, experiencing changes in perception (Cosman and Vecera, 2010), attention (e.g., Reed et al., 2006; Abrams et al., 2008; Davoli and Brockmole, 2012) and memory (Tseng and Bridgeman, 2011; Thomas et al., 2013) (see Brockmole et al., 2013 and Tseng et al., 2012, for reviews). Investigators have suggested these changes in visual processing near the hands may assist observers in evaluating items that are candidates for action by enhancing analysis (Abrams et al., 2008; Tseng and Bridgeman, 2011; Davoli et al., 2012a), biasing for item-specific detail (Davoli et al., 2012b), altering representations of space via bimodal visuotactile neurons (Reed et al., 2006), or shifting information processing toward the action-oriented magnocellular visual pathway (Gozli et al., 2012).

The flurry of recent research on changes in visual processing of objects near the hands appeals to action-based explanations for why these changes occur, which raises the possibility that varying the affordances for action in a given situation may influence nearby-hand effects on vision. Observers detect targets more quickly when they are presented near the palm of the hand than the back of the hand or forearm, suggesting that processing changes occur specifically within the hands' grasping space (Reed et al., 2010). Similar results occur for targets presented near the functional end of tools (Kao and Goodale, 2009; Reed et al., 2010; Brown et al., 2011; Gozli and Brown, 2011), backing the notion that alterations in visual processing are tied to the potential for producing effective action. In addition, nearby-hand effects tend to drop off as the distance between the hands and the relevant visual stimulus increases (Tseng and Bridgeman, 2011; Adam et al., 2012). While this work points to the ties between action affordances and changes in visual processing near the hands, previous investigations have focused almost exclusively on comparing performance in visual paradigms under conditions in which participants either take hold of a display with both hands (e.g., Abrams et al., 2008) or hold a single open hand on one side of the display (e.g., Reed et al., 2006) against performance in conditions in which both hands are positioned away from the display. In these popular variations, observers position their hand(s) in a manner that affords a power grasp: the fingers function as a unit that can curl around an object to secure it against the palm. Although a few studies have also documented nearby-hand effects when observers position their hands below a display (Lloyd et al., 2010; Tseng and Bridgeman, 2011; Adam et al., 2012), even in these cases, the hands remain in a position with the fingers extended and held together.

Human hands possess a range of motion and action capabilities, from the rapid coordination of a rugby player catching a pass to the precision of a tailor threading a needle. However, investigations of changes in visual processing near the hands have essentially only examined effects associated with the power grasp hand posture. A one-size-fits-all approach to nearby-hand effects on vision ignores the possibility that the posture of the hands themselves—and the actions these postures afford—could potentially shape visual processing biases near the hands. Recent findings suggest that planning and preparing a particular action biases selection of action-congruent features in visual search (Wykowska et al., 2009). Similarly, viewing photographs of hands in particular postures can prime responses to objects that afford grasps of the same posture (Borghi et al., 2007). Such action-specific effects suggest that hand posture may also influence changes in visual processing near the hands.

As a first step in investigating the influence of changes in hand posture on nearby-hand vision effects, I asked participants to perform a visual task not only under the typical open palm posture that affords power grasps, but also under a complimentary pincer posture that affords precision grasps (see Figure 1). Depending on the activity intended, people typically adopt one of these two postures to grip nearby objects—flexing the fingers around an object to hold it against the palm in a power grasp or securing an object between the pads of the thumb and fingers in a precision grasp (Napier, 1956). Observers represent objects based on whether they more readily afford power or precision grasps (Tucker and Ellis, 2001, 2004), and viewing pictures of hands in these two different grasp postures automatically biases attention to grasp-congruent objects in a display (Fischer et al., 2008). People have extensive experience using both power and precision grasps and presumably associate each grasp posture with the specific class of actions it affords, making a comparison of these two postures a solid test case of the hypothesis that the affordances of hand postures modulate nearby-hand effects on vision.

To examine the influence of grasp posture on visual processing near the hands, I employed the classic attentional orienting paradigm (Posner et al., 1987) that Reed et al. (2006) used in their seminal paper on nearby-hand effects. In the original work, Reed et al. (2006) asked participants to detect the appearance of a visual target that could appear either to the right or left of a central fixation cross. In some conditions, participants placed either their left hand next to the left-side target location or their right hand next to the right-side target location with the open palm facing the target. The authors found that participants were faster to detect targets appearing near their hands than targets away from their hands, suggesting that attention is prioritized to the space near the hands (Reed et al., 2006). Additional experiments replicated and extended these findings, showing that facilitation occurs for targets appearing near the open palm of the hand—targets positioned with respect to the hand in a manner affording a power grasp—but not for targets near the back of the hand (Reed et al., 2010). The experiments presented here investigate this attentional prioritization effect, examining whether prioritization of space near the hands is dependent upon grasp posture. While I find evidence for attentional prioritization of the space near the hands when observers position their hands for a power grasp, when they instead position their hands for a pincer grasp, this prioritization effect disappears.

## Experiment 1

In order to investigate the influence of grasp posture on attentional prioritization of the space near the hands, it is prudent to first replicate the original finding that observers are faster to detect targets near a hand positioned on the screen in a relaxed power grasp posture—thumb side up, fingers held together in a single unit—than targets far from the hand. I compared participants' performance on the standard covert attention task under conditions in which they held their left or right hand on the display in this posture against conditions in which participants instead rested one of their hands in their laps.

**Figure 1:**
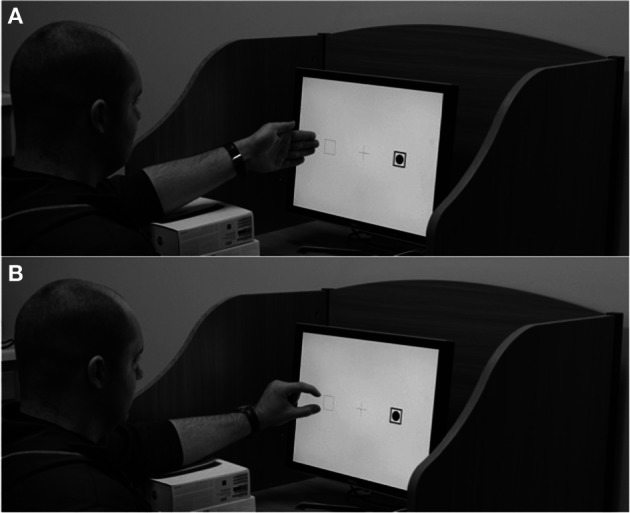
**Grasp postures in (A) the palm conditions of Experiments 1 and 3 and (B) the pinch conditions of Experiments 2 and 3**.

## Methods

### Participants

Twenty-five right-handed undergraduate volunteers from Vanderbilt University participated for course credit. The Vanderbilt University Institutional Review Board approved the experimental protocol and all participants provided informed consent.

### Stimuli and apparatus

Stimuli were presented on a color monitor set at a resolution of 1024 × 768 pixels and a refresh rate of 89 Hz. Participants sat approximately 55 cm from the monitor. All stimuli were black presented on a white background. The stimuli consisted of a fixation cross (3°), two empty squares (3°) that were 6° to the left and right of fixation, and a target dot (2.3°). Participants made responses with a standard keyboard.

### Procedure and design

Participants performed an attentional orienting target detection task designed to match the conditions of the original Reed et al. (2006) study. Each trial began with the presentation of a central fixation cross flanked by an empty square to the left and an empty square to the right. After a random delay between 1500 and 3000 ms, the border of one of the two squares darkened, serving as a cue to the target location. On 70% of trials, this cue was valid and a solid target dot appeared in the cued square 200 ms later and remained on the display until participants indicated they had detected the target by pressing the “h” key on the keyboard. On 20% of trials, the cue was invalid and the target instead appeared in the opposite square and again remained on the display until participants made a detection response. The remaining 10% of trials served as catch trials in which no target dot appeared; on these trials, the cue display remained onscreen for 3000 ms before the next trial began.

Participants performed blocks of trials in four hand posture conditions. In the *left-rest* condition, participants responded with their right hand and rested the free left hand in their laps. In the *right-rest* condition, participants responded with their left hand and rested their right hand in their laps. In the *left-palm* condition, participants again responded with their right hand, but extended their left hand to rest against the display next to the left target position. Finally, in the *right-palm* condition, participants responded with the left hand and extended the right hand to rest against the display next to the right target position. For the palm conditions, participants held their hands with the fingers together, thumb side up, with their palms facing toward the center of the screen in the same relaxed position affording a power grasp employed in previous investigations (Reed et al., 2006, 2010). Before a block of trials in the palm conditions began, participants viewed a display with written instructions about hand placement, the target boxes and fixation cross, and three small filled dots (0.5°) arranged in a vertical line subtending 3.5° placed 1.5° to the side of one target box that served as a guide to help them position the hand in a consistent location on the display. These guide dots were removed before the first trial in a block began. Before each block of trials in the rest conditions, participants viewed a display showing written instructions about hand placement as well as the target boxes and fixation cross. In the palm conditions, participants rested their extended arm on a brace to minimize discomfort. Participants performed a short block of 20 practice trials in the left-rest condition before completing two blocks of 60 trials each in the four hand conditions for a total of 480 trials. Both trials within a block and hand conditions across blocks were presented in a randomized order.

## Results and discussion

**Figure 2:**
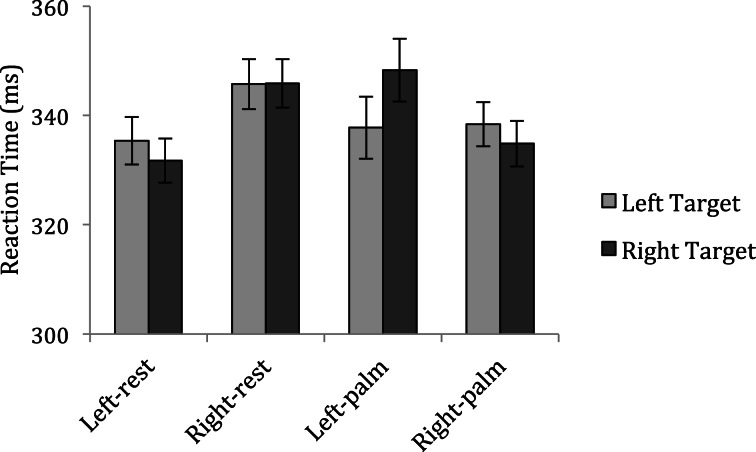
**Mean reaction times to detect targets in Experiment 1**.

One participant made excessive response errors on catch trials (>50%) and was excluded from analyses. The remaining 24 participants incorrectly made a response on an average of 11.73% of catch trials; data for these trials were not analyzed. To eliminate anticipation and inattention errors, trials with a reaction time of less than 100 ms or greater than two standard deviations from a participant's mean reaction time were also excluded from the analyses. Mean reaction times were calculated to targets on the right and left side under each of the four hand conditions^1^ and are displayed in Figure 2.

If participants prioritize the space near the hand as previous research suggests (Reed et al., 2006, 2010), then participants should be facilitated in detecting targets appearing on the same side as their hand in the palm conditions. A repeated measures analysis of variance with factors of Hand Posture (rest vs. palm), Response Hand (left vs. right) and Target Side (left vs. right) showed that participants tended to be faster making responses with their dominant hand than their non-dominant hand when in the rest conditions: Hand Posture × Response Hand interaction, *F*_(1, 23)_ = 4.84, *p* = 0.038. More importantly, participants were also relatively faster to detect targets appearing near a hand positioned in the palm posture that affords a power grasp than targets appearing away from a hand: Hand Posture × Response Hand × Target Side interaction, *F*_(1, 23)_ = 4.07, *p* = 0.056. To interpret this marginally significant 3-way interaction, separate ANOVAs with factors of response hand and target side were conducted for each hand posture. These analyses confirmed that the interaction between response hand and target side was significant in the palm conditions, *F*_(1, 23)_ = 4.77, *p* = 0.039 (left-palm condition: left targets = 338 ms vs. right targets = 348 ms; right-palm condition: left targets = 338 ms vs. right targets = 335 ms), but not the rest conditions, *F*_(1, 23)_ < 1, *ns* (left-rest condition: left targets = 335 ms vs. right targets = 332 ms; right-rest condition: left targets = 346 ms vs. right targets = 346 ms). These *post-hoc* analyses also showed a main effect of response hand for the rest conditions, *F*_(1, 23)_ = 7.75, *p* = 0.011. No other main effect or interaction approached significance (all *p*-values > 0.1).

The results of Experiment 1 serve as an independent replication of the attentional prioritization effects previously documented by Reed and her colleagues (2006, 2010). When participants held their hand on the display in a posture that affords a power grasp, they were facilitated in detecting targets that appeared next to their open palm. However, the side on which a target appeared had no influence over reaction times when participants instead held their free hand in their laps. Having thus confirmed the reproducibility of the original attentional prioritization findings, in Experiment 2 I investigated whether observers also prioritize the space near their hands when they adopt a pincer grasp posture.

## Experiment 2

Recent studies examining visual processing near the hands seem to suggest that, as long as visual stimuli appear within the hands' functional space, nearby-hand effects should occur. Yet the hands can serve multiple functions, and observers' experiences may create biases toward visual information that is relevant for different types of actions (e.g., Wykowska et al., 2009). To investigate the possibility that hand posture influences visual processing biases, in Experiment 2, I asked participants to perform the same covert orienting task while holding one hand next to a target location on the display, but in this case, they positioned their hand in a pincer posture in which the thumb and forefinger rested near the target location, affording a precision grasp. As in the previous experiment, this posture places targets near the hand within its functional grasping space. However, the type of grasp that observers were positioned to perform is quite different, and this is a difference to which the visual system is attuned (Tucker and Ellis, 2001, 2004; Fischer et al., 2008). In Experiment 2 participants were essentially at the ready for a precise action instead of a power action. If proximity to the functional space of a hand alone facilitates target detection, then participants in Experiment 2 should show the same effect of attentional prioritization near the hands as did participants in Experiment 1. However, if grasp posture has an influence on visual processing near the hands, then the pattern of data for Experiment 2 may differ from the near-hand facilitation effect I found in the first experiment.

## Methods

### Participants

Twenty-four right-handed undergraduate volunteers from North Dakota State University participated for course credit. The North Dakota State University Institutional Review Board approved the experimental protocol and all participants provided informed consent.

### Stimuli, apparatus, procedure, and design

Stimuli identical to those used in Experiment 1 were presented on a color monitor set at a resolution of 1024 × 768 pixels and a refresh rate of 75 Hz. Participants again sat approximately 55 cm from the monitor and performed the target detection task from Experiment 1. They performed blocks of trials in four hand posture conditions, two of which were replications of the rest conditions in Experiment 1, with identical instruction screens at the beginning of each block. In addition, participants performed the attentional orienting task under two new hand posture conditions in which they rested a hand against the display in a pincer grasp posture. In the *left-pinch* condition, participants responded with their right hand on the keyboard and extended their left hand to rest against the display next to the left target position. In the *right-pinch* condition, participants responded with the left hand and rested the right hand against the display next to the right target position. For the pinch conditions, instead of extending their hands with the fingers together, participants held their thumb and forefinger next to an empty square, positioned in a manner affording precision grasps (see Figure 1B). Before a block of trials in the pinch condition began, participants viewed a display with written instructions about hand placement, the target boxes and fixation cross, and two filled dots arranged vertically—one slightly above the target box, the other slightly below, again subtending 3.5°—that served as a guide indicating where the thumb and forefinger should rest during the upcoming trials. In these conditions, participants rested their extended arm on a brace to minimize discomfort. Participants performed a short block of 20 practice trials in the left-rest condition before completing two blocks of 60 trials each in the four hand conditions—480 trials total—in a randomized order.

## Results and discussion

**Figure 3:**
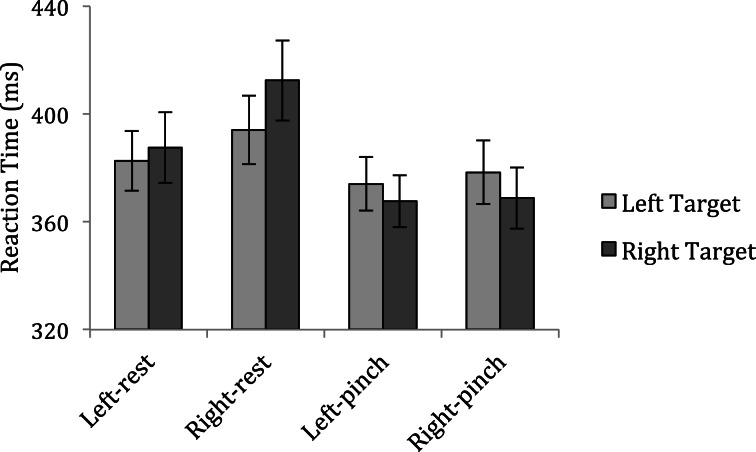
**Mean reaction times to detect targets in Experiment 2**.

Participants incorrectly made a response on an average of 17.97% of catch trials for which data were not analyzed. Anticipation and inattention errors were excluded from analyses using the same criteria as in Experiment 1. Figure 3 displays mean reaction times across conditions for Experiment 2.

If observers prioritize the space near the hands—regardless of the hands' posture—then participants should again be faster to detect targets appearing near the hand than away from the hand in the pinch conditions. A repeated measures analysis of variance with factors of Hand Posture (rest vs. pinch), Response Hand (left vs. right) and Target Side (left vs. right) showed only that participants tended to respond more quickly in the pinch conditions than the rest conditions: main effect of Hand Posture, *F*_(1, 23)_ = 5.30, *p* = 0.031, possibly indicating greater overall arousal when a hand was held on the display in a precision posture^2^ (left-pinch condition: left targets = 374 ms vs. right targets = 368 ms; right-pinch condition: left targets = 378 ms vs. right targets = 369 ms; left-rest condition: left targets = 383 ms vs. right targets = 388 ms; right-rest condition: left targets = 394 ms vs. right targets = 412 ms). No other main effect or interaction approached significance (all *p*-values > 0.1).

Although participants in Experiment 2 performed the same target detection task as participants in Experiment 1, unlike participants in the first experiment, these observers were no faster to detect targets appearing near their hands than targets appearing away from their hands. The pattern of performance in the pinch condition was similar to the pattern of performance in the rest condition, suggesting that, at least from a target location standpoint, there was no difference between performing the task while a hand was on the display than when it rested in a participant's lap. A difference in hand proximity across experiments cannot explain this pattern of results: participants in Experiment 2 held their hands just as close to the target locations as participants in Experiment 1. Likewise, the lack of attentional prioritization of the space near the hands cannot be due to the fact that targets appeared outside of functional hand space: as in Experiment 1, near-hand targets in Experiment 2 appeared within the hands' grasping space. Instead, the different pattern of results across these two experiments must be a function of the different grasp postures participants adopted while performing the task. Holding the hand in an open palm posture that affords a power grasp creates an attentional bias to the space near the hand, but observers who held their hands in a pincer posture that affords a precision grasp were no more likely to attend to locations near their hand than locations away from their hand.

## Experiment 3

Although the pattern of results across the first two experiments suggests that attentional prioritization occurs for the space near hands positioned for a power, but not a precision grasp, it is difficult to firmly conclude that grasp posture influences the allocation of attention near the hands without directly comparing the two grasps in a single study. In other words, the fact that participants showed significant facilitation in detecting targets near the hands in Experiment 1, but no significant facilitation in Experiment 2 does not necessarily imply the difference between performance under power grasp and precision grasp postures is itself significant. I address this issue in Experiment 3 by asking a single group of participants to perform the attentional orienting task under both the palm conditions of Experiment 1 and the pinch conditions of Experiment 2. If grasp posture modulates attentional prioritization near the hands, then participants in this experiment should be facilitated in detecting targets near the hand in the palm conditions, but not the pinch conditions.

## Methods

### Participants

Twenty-nine right-handed undergraduate volunteers from North Dakota State University participated for course credit. The North Dakota State University Institutional Review Board approved the experimental protocol and all participants provided informed consent.

### Stimuli, apparatus, procedure, and design

Experiment 3 was a replication of the previous two experiments combining the palm and pinch conditions of Experiments 1 and 2. Following a short block of 20 practice trials in which participants responded with the right hand and held their left hand in their laps, participants performed two blocks of 60 trials each of the left-palm, right-palm, left-pinch, and right-pinch conditions—a total of 480 trials—in a randomized order.

## Results and discussion

**Figure 4:**
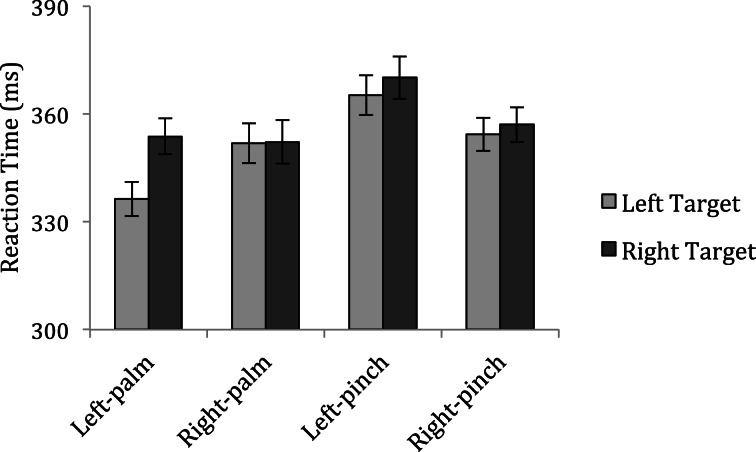
**Mean reaction times to detect targets in Experiment 3**.

Five participants made excessive response errors on catch trials (>50%) and were excluded from analyses. The remaining 24 participants incorrectly made a response on an average of 19.02% of catch trials for which data were not analyzed. Anticipation and inattention errors were excluded from analyses using the same criteria as in the previous experiments. Figure 4 displays mean reaction times across conditions for Experiment 3.

To look for supporting evidence that grasp posture modulates attentional prioritization of the space near the hands, I conducted a repeated measures analysis of variance with factors of Hand Posture (palm vs. pinch), Response Hand (left vs. right) and Target Side (left vs. right). A significant interaction between hand posture, response hand, and target side, *F*_(1, 23)_ = 4.71, *p* = 0.041 mediated main effects of hand posture, *F*_(1, 23)_ = 8.54, *p* = 0.008, and target side, *F*_(1, 23)_ = 6.89, *p* = 0.015, and interactions between posture and response hand, *F*_(1, 23)_ = 6.84, *p* = 0.016, and response hand and target side, *F*_(1, 23)_ = 6.75, *p* = 0.016. An examination of Figure 4 indicates that a facilitation of responses near the left hand in the palm condition coupled with a lack of difference between responses to targets near vs. away from the hands in the pinch conditions drove the significant three-way interaction. Separate *post-hoc* analyses for each hand posture confirm this impression: while there was a significant main effect of target side, *F*_(1, 23)_ = 12.15, *p* = 0.002 mediated by a significant interaction between response hand and target side, *F*_(1, 23)_ = 8.84, *p* = 0.015 in the palm conditions (left-palm condition: left targets = 336 ms vs. right targets = 354 ms; right-palm condition: left targets = 352 ms vs. right targets = 352 ms), only the main effect of response hand was significant in the pinch conditions, *F*_(1, 23)_ = 7.00, *p* = 0.014, with participants being somewhat slower to respond with their dominant right hand when their left hand was on the display (left-pinch condition: left targets = 365 ms vs. right targets = 370 ms; right-pinch condition: left targets = 354 ms vs. right targets = 357 ms). No other main effect or interaction approached significance (all *p*-values > 0.1).

The results of Experiment 3 provide a replication of the key findings of the first two experiments: participants were once again faster to respond to targets appearing near the hand in the palm conditions, but did not show the same facilitation when targets appeared near the hand in the pinch conditions. Again, although the targets appeared in the hands' grasping space in both the palm and pinch conditions and participants held their hands equally close to the target locations under both postures, they only prioritized targets near their hands when the hands were positioned to afford a power grasp. Importantly, the interaction between hand posture, response hand, and target side was significant, providing a direct contrast between nearby-hand effects under a power vs. precision grasp posture.

## General discussion

Nearby-hand effects on vision may reflect adaptations that allow observers to determine appropriate responses to nearby objects by processing these candidates for action differently than more distant stimuli (Graziano and Cooke, 2006; Abrams et al., 2008; Brockmole et al., 2013). The majority of work investigating these effects has focused on how the presence of the hands, in general, influences processing. The results of the current study suggest that the presence of the hands will not always necessitate the same specific alterations in processing, but instead that biases in visual processing near the hands can change when observers position their hands to afford different types of actions.

Across three experiments, I found evidence that attentional prioritization near the hands relies upon the hands' posture. While participants in Experiments 1 and 3 demonstrated a clear attentional bias toward locations near their hands, detecting targets more quickly when they appeared within power grasping space^2^, participants in Experiments 2 and 3 who instead held their hands in a pincer grasp posture did not favor targets appearing on one side of the display over another. In other words, observers prioritized their attention to the hands' functional grasping space, but only when the hands were at the ready for a power, and not a precision, grasp. These results suggest that changes in visual processing near the hands rely not only on the relationship between the hands' location and the relevant visual information (i.e., Reed et al., 2010; Adam et al., 2012), but also the potential actions that the hands' positioning affords.

Why would observers be biased to attend to the space near their hands when in a power grasp posture, but not when in a precision grasp posture? If adaptations for effective action production drive nearby-hand effects, then the influence the hands exert over visual processing should be modulated by the context between the nature of the processing task and stimuli and the hands' affordances for action. Although the data suggest that participants were no more biased to attend to one side of a display when their hands were in the pinch posture than they were when their hands were in their laps, this does not necessarily imply that precision grasping hands will never alter visual processing. Bimodal neurons show selectivity based on observed object size and power vs. precision grasps (e.g., Fadiga et al., 2000), raising the possibility that representations related to different hand postures may lead to different visual biases. The precision grasp posture may have been ill suited to meet the demands of the attentional orienting task, but perhaps a more detail-oriented visual task could be more compatible with this posture. The visual biases that aid a rugby player in catching a pass may differ substantially from those that help a tailor thread a needle.

A recent finding in the literature on nearby-hand effects may speak to this possibility. Gozli et al. (2012) found that observers were better at a temporal gap detection task when they grasped the display between both hands than when they kept their hands away from the display, but were better at a spatial gap detection task when their hands were far from the display. The authors suggest that placing the hands near an object biases visual information processing toward increased contributions from the high temporal, low spatial resolution magnocellular pathway (Pokorny and Smith, 1997; Maunsell et al., 1999), while keeping the hands away from an object biases contributions from the high spatial, low temporal resolution parvocellular visual pathway (Derrington and Lennie, 1984; Pokorny and Smith, 1997). Gozli et al. (2012) argue these results are consistent with a framework in which observers prioritize action when processing objects near the hands, but prioritize perception when processing objects far from the hands. Data from the palm conditions of the current study are in line with this theory: the power grasping posture facilitated a target detection task requiring rapid responses to large changes in luminance to which the magnocellular pathway is sensitive. However, although the precision grasp posture does afford an action, instead of creating a bias toward the action-oriented magnocellular pathway, it may have introduced bias toward the more detail-oriented parvocellular pathway. The precision grasp posture affords actions that bring nearby objects into contact with the pads of the thumb and forefingers, areas of the hand with high tactile spatial acuity that aid in delicate work, while the power grasp posture affords actions that bring objects into contact with the base of the fingers and palm, areas of the hand with lower tactile sensitivity that enable faster and more forceful work (Johansson and Vallbo, 1979; Craig, 1999; Craig and Lyle, 2001). An observer who is prepared to perform a power grasp is ready to make a quick action, as in the case of a rugby player catching a pass. On the other hand, an observer who is at the ready to interact with an object using a precision grasp, such as a tailor threading a needle, may benefit from a more fine-grained analysis of the object's visual properties. The findings of the current study are consistent with the theory that power grasps bias processing toward contributions from the magnocellular pathway. Future work will be necessary to explore the notion that precision grasps may enhance parvocellular contributions.

The results of the current study provide the first evidence that changes in visual processing near the hands may rely on the hands' grasping posture. As work investigating nearby-hand effects on vision moves forward, it is important to consider not only how the hands' presence can alter processing, but also how the hands' potential for actions may modulate these effects. An examination of the influence of hand posture on processing biases may ultimately point the way toward enhanced understanding of the underlying mechanisms of vision near the hands.

### Conflict of interest statement

The author declares that the research was conducted in the absence of any commercial or financial relationships that could be construed as a potential conflict of interest.

## Footnotes

^1^Previous investigations have found that cue validity does not influence attentional prioritization of space near the hand (Reed et al., 2006, 2010). I have chosen to collapse the data across valid and invalid trials to simplify presentation of results. The pattern of data was similar across both valid and invalid trials in all experiments.

^2^While fMRI evidence shows that executing both power and precision grasps activates primary motor cortex, precision grasps are also associated with more widespread bilateral BOLD activity in premotor and parietal areas, suggesting that a precision grasp is more demanding in terms of neural control (Ehrsson et al., 2000). The pinch hand posture may therefore be more arousing compared to a baseline rest condition than the power hand posture.
